# Supplementary material for: A room temperature continuous-wave nanolaser using colloidal quantum wells
Source: Nat Commun. 2017 Jul 26;8:143. doi: 10.1038/s41467-017-00198-z (PMC5529567; doi:10.1038/s41467-017-00198-z)
Supplement: Supplementary file 1 — Supplementary Information [file 41467_2017_198_MOESM1_ESM.pdf]

File name: Supplementary Information
Description: Supplementary Figures, Supplementary Notes and Supplementary References

File name: Peer Review File
Description:

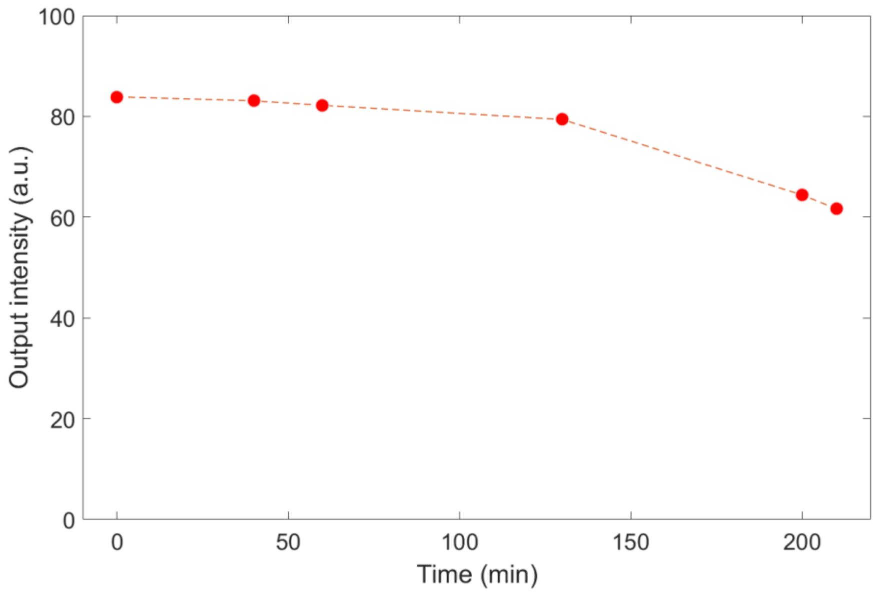

Supplementary Figure 1. | Device photostability. Device output intensity from Lorentzian fit of the cavity mode as a function of time.

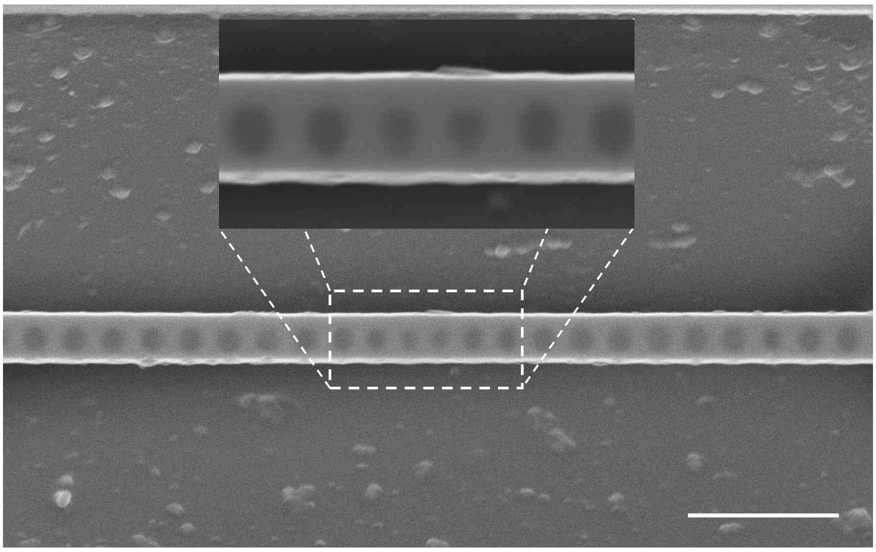

Supplementary Figure 2. | Deposition of nanoplatelets. Scanning-electron-microscope image of a nanobeam cavity after deposition of nanoplatelets from solution. Scale bar is 1 μm. The inset shows the centre region indicated by the dashed square at higher magnification. The semitransparent area of holes indicates that the nanoplatelet film uniformly covers the entire nanobeam cavity.

Supplementary Note 1: Coupling efficiency estimation

We define the Purcell factor as $F = \frac{\tau_{\text{sub}}}{\tau_{\text{c}}} = \frac{\gamma_{\text{c}}}{\gamma_{\text{sub}}}$, where τ_{sub} is the lifetime of nanoplatelets on the un-patterned substrate, τ_{c} is the lifetime of nanoplatelets coupled to the cavity, $\gamma_{\text{c}} = \frac{1}{\tau_{\text{c}}}$ is the decay rate of a nanoplatelet in the cavity, and $\gamma_{\text{sub}} = \frac{1}{\tau_{\text{sub}}}$ is the decay rate on the un-patterned substrate. The spontaneous emission coupling efficiency is given by $\beta = 1 - \gamma_{\text{leak}} / \gamma_{\text{c}}$, where $\gamma_{\text{c}} = \gamma_{\text{cav}} + \gamma_{\text{leak}}$; here, γ_{cav} is the nanoplatelet decay rate into the cavity mode and γ_{leak} is the decay rate of the nanoplatelets into all other radiative and non-radiative channels. An emitter inside of a cavity will typically experience Purcell enhancement when coupled to the cavity,[2] and Purcell suppression when decoupled (either due to detuning or poor spectral matching with the cavity),[3] which means that $\gamma_{\text{leak}} < \gamma_{\text{sub}}$. In this case $F < \frac{\gamma_{\text{c}}}{\gamma_{\text{leak}}}$ and thus $\beta > 1 - 1/F$.

Supplementary Note 2: Rate equation analysis

To extract the spontaneous emission coupling efficiency β and the threshold P_{th} of the laser, we use a standard coupled rate-equation model for the carrier density N and the cavity photon number p of a semiconductor laser diode, modified from the work by G. Björk and Y. Yamamoto.[1] We use the coupled rate equations

$$\frac{d}{dt}N = \frac{\eta_{\text{in}} P_{\text{in}}}{\hbar\omega_{\text{p}} V} - \frac{N}{\tau_{\text{sp}}} - \frac{N}{\tau_{\text{nr}}} - \frac{gp}{V} \tag{1}$$

$$\frac{d}{dt}p = -(\gamma - g)p + \frac{\beta V}{\tau_{\text{sp}}}N \tag{2}$$

where P_{in} is the optical power of the pump laser, η_{in} is the fraction of incident optical pump power absorbed by the gain material, $\hbar\omega_{\text{p}}$ is the photon energy of the pump laser, V is the volume of the gain medium, τ_{sp} and τ_{nr} are the exciton radiative and nonradiative

lifetimes respectively, and $g = g'(N - N_0)$ is the material gain, assumed to be linearly proportional to the carrier density, where $g' = \beta V / \tau_{\mathrm{sp}}$ is a material constant and N_0 is the transparency carrier density of the material. The cavity photon number is $p = P_{\mathrm{out}} / \hbar\omega\gamma\eta_{\mathrm{out}}$, where P_{out} is the output power onto the detector, ω is the cavity resonance frequency, η_{out} is the laser output collection efficiency, $\gamma = \omega / Q$ is the cavity decay rate, and β is the spontaneous emission coupling efficiency. The detected electron number on the CCD is linearly proportional to the output power ($n_{\mathrm{ccd}} = kP_{\mathrm{out}}$). Thus, we have $p = \alpha n_{\mathrm{ccd}}$, where $\alpha = 1 / k\hbar\omega\gamma\eta_{\mathrm{out}}$.

The steady-state solution to the above rate equations is

$$P_{\mathrm{in}} = \frac{\hbar\omega\gamma}{\beta\eta_{\mathrm{in}}}\left[\frac{p}{1+p}\left(1+\xi\right)\left(1+\beta p\right) - \xi\beta p\right] \tag{3}$$

where $\xi \equiv \dfrac{N_0 \beta V}{\gamma\tau_{\mathrm{sp}}}$ is the cavity photon number at transparency. We substitute $p = \alpha n_{\mathrm{ccd}}$ into the cavity photon number and treat β, ξ, η_{in} and α as fitting parameters. From the fit, we obtain $\beta = 0.81 \pm 0.03$, and $\eta_{\mathrm{in}} = 21.6 \pm 0.4\%$. We obtain the lasing threshold by setting $p = 1$ in Supplementary Equation 3 which gives $P_{\mathrm{th}} = P_{\mathrm{in}}\big|_{p=1} = 0.97 \pm 0.03\ \mu\mathrm{W}$.

Supplementary Note 3: Device photostability

To demonstrate the stable performance of our lasing device, we conduct a measurement on another lasing device prepared by the same method as the measured one in the main text. The pump power is kept constant at 10 µW, which is about 10 times above the threshold. From the Lorentzian fit of the acquired spectrum, we find cavity *Q* of 4,900 and a resonance wavelength of 664.5 nm at the beginning of our experiment. We record the output intensity as a function of time while the device is continually excited for 3.5 hours. As shown in Supplementary Figure 1, the output power from the device drops about 20 % compared to its initial value during this time period, which is likely due, at least in part, to drift in the collection

efficiency for our measurement system.

Supplementary References

1 Björk, G. & Yamamoto, Y. Analysis of semiconductor microcavity lasers using rate equations. *IEEE J. Quant. Electon.* **27**, 2386-2396 (1991).

2 Pelton, M. Modified spontaneous emission in nanophotonic structures. *Nat. Photon.* **9**, 427-435 (2015).

3 Gérard, J. *et al.* Enhanced spontaneous emission by quantum boxes in a monolithic optical microcavity. *Phys. Rev. Lett.* **81**, 1110-1113 (1998).
